# Impact of a Dedicated Trauma Consult Service on Burnout Among Physicians and Nurses: A Longitudinal Survey

**DOI:** 10.7759/cureus.104108

**Published:** 2026-02-23

**Authors:** Robert Green, Darby Green, Adam Harris, Breanne Gillis, Sarah Sturge, Daniel Cashen, Hillary Ferguson, Izabella Opra

**Affiliations:** 1 Trauma Nova Scotia, Nova Scotia Health, Halifax, CAN; 2 Emergency Medicine, Dalhousie University, Halifax, CAN; 3 Bioethics, Dalhousie University, Halifax, CAN

**Keywords:** professional burnout, survey methodology, trauma care system, trauma consultant, workload and burnout

## Abstract

Background

Trauma and emergency medicine providers have previously reported high levels of burnout, which were exacerbated by the COVID pandemic. This study evaluated the impact of trauma system change with the introduction of a novel Trauma Consult Service (TCS) on clinician burnout levels at zero, six, and 12 months for major trauma patients in Nova Scotia.

Methods

A longitudinal electronic survey was developed and piloted by the research team. Baseline surveys were administered in November 2022 to clinicians who pivoted from their usual career to a dedicated focus on inpatient trauma care. Demographic data and burnout symptoms were assessed using surveys, i.e., the Copenhagen Burnout Inventory, the Maslach Burnout Inventory, and the Utrecht Work Engagement Scale. Follow-up surveys were conducted at six and 12 months. Generalized estimating equations (GEE) were fitted to determine the effect of the Trauma Consult Service on burnout symptoms during the inaugural year of the service.

Results

A total of 29 survey responses were received from Trauma Consult Physicians (n=13), Trauma Team Leaders (n=9), and Trauma Consult Nurses (n=7). Nearly all were full-time employees (96.6%), and most had 6-10 years of experience providing trauma care (58.6%). At the one-year follow-up, there were reductions in the Copenhagen Burnout Inventory, which measures personal and work-related burnout, as well as in the Maslach Burnout Inventory's single-item measures for emotional exhaustion and depersonalization. Generalized estimating equation models revealed increased staff engagement across all Utrecht Work Engagement Scales. Notably, few participants reported using mental health resources to improve their well-being (3.4%).

Conclusion

Burnout symptoms among physicians and nurses were reduced at one year post-implementation of a dedicated inpatient Trauma Consult Service. Further investigation is warranted to understand how system-level changes impact burnout. These findings suggest that Canadian emergency medicine providers could benefit from exploring system-level role modifications, such as dedicated trauma services, as a potential strategy to mitigate clinician burnout and enhance workforce engagement.

## Introduction

Burnout is a syndrome that has been recognized as an acute and chronic occupational stress response in medical providers, particularly in high-pressure specialties, including emergency medicine and trauma. Although burnout may manifest in various ways, it is most often characterized as a stress response that includes three components: emotional exhaustion, depersonalization, and a sense of reduced personal accomplishment [1]. Recent investigations have highlighted that the burdens of limited staffing and high turnover rates may result in moral distress and shorten career duration [2,3]. The demanding nature of the emergency medicine environment, characterized by extended work hours, administrative burdens, and an unpredictable, emotionally intense pace of patient care, increases the risk of burnout among physicians and other clinicians [4,5]. The prevalence of burnout increased among physicians and allied healthcare providers during the COVID pandemic, adding additional strain to an overburdened healthcare system [6,7]. Many clinicians experienced the very real costs of burnout (e.g., anxiety, addiction, attrition, or thoughts of suicide), which negatively affected patient care and challenged the healthcare system across Canada [8].

As part of the Nova Scotia Provincial Trauma Program, the launch of a novel inpatient Trauma Consult Service (TCS) in 2022 offered a unique opportunity to investigate the impact on physician and nurse burnout. The TCS, comprising Trauma Team Leaders from emergency medicine, critical care, and anesthesia staff, collaborates with a trauma resource nurse (RN) to provide comprehensive care for trauma inpatients throughout the duration of their hospital stay, from the time of trauma team resuscitation to patient discharge. The TCS delivers comprehensive education to inpatient and outpatient units, ensuring the continuity of care for trauma patients.

We hypothesized that clinicians who were allowed to modify part of their clinical focus from their base specialty (i.e., emergency medicine, anesthesia, and critical care) to include dedicated weeks of service within the novel TCS would experience a decrease in burnout symptoms. We investigated burnout rates among Trauma Team Leaders and TCS members at zero months (baseline), six months, and 12 months following the implementation of the service to determine whether well-being and symptoms of burnout would improve over time. Investigations that focus on the impact of occupational stress (such as burnout syndrome) are necessary to determine future interventions aimed at supporting healthy physicians and nurses in emergency medicine, trauma, and other high-stress medical specialties.

This article was previously presented as a poster at the 2024 London Trauma Conference, in London, England, from December 3rd to 6th 2024, and at the 2025 Trauma Association of Canada Annual Scientific Meeting and Congress in Gatineau, Quebec, from March 19th to 21st 2025.

## Materials and methods

Study setting

The Canadian province of Nova Scotia has an inclusive trauma system where patients receive care from a dedicated trauma team and have early access to specialty consultation. In Nova Scotia, there are two level 1 trauma centers (one adult and one pediatric) and eight regional level 3 trauma centers throughout the province. All patients with major trauma are transported to the level 1 trauma center in the Queen Elizabeth II Health Sciences Centre (QEII) in Halifax, Nova Scotia.

Survey design

The study was approved by the Nova Scotia Health Research Ethics Board (approval number: 1028409). We developed and tested an electronic survey using SelectSurvey version 5.0 (ClassApps, Apollo Beach, FL) (see Appendices). This is an exploratory survey that used three different assessment tools to measure burnout. The survey was administered via email at three different data collection points: zero months (November 2022), six months (May 2023), and 12 months (November 2023). Subsequently, one reminder email was distributed within 10 days after each time point. The study was conducted in accordance with the CROSS checklist for reporting survey research [9].

Outcome measures

Data collected included demographics and burnout symptoms. Demographic questions included work experience and employment type. The participants were asked multiple questions about work-related impacts, such as negative behaviors they had experienced or witnessed while working with the Trauma Consult Service (TCS), and whether they used wellness resources.

We evaluated burnout symptoms using three previously validated scoring systems: the Copenhagen Burnout Inventory, the Maslach Burnout Inventory single-item scales, and the Utrecht Work Engagement Scale. The Copenhagen Burnout Inventory measures personal, work-related, and patient-related burnout using a 19-item survey [10]. Multiple questions were included for each of the subscales (personal, work-related, and patient-related burnout). Responses were on five-point scales, by either frequency (e.g., "never" to "always") or intensity (e.g., "to a very low degree" to "to a very high degree"), and were coded into scores of "one, two, three, four, and five" for modelling. Scores of less than two indicate low levels of burnout, and scores greater than or equal to three indicate a high degree of burnout.

The Maslach Burnout Inventory measures work-related burnout on two single-item subscales: emotional exhaustion and depersonalization [11,12]. This inventory was used with licensed permission. For this study, only emotional exhaustion and depersonalization were assessed. Each subscale includes a seven-point Likert-type frequency response scale (zero, never; one, a few times a year or less; two, once a month or less; three, a few times a month; four, once a week; five, a few times a week; and six, every day). Scales are scored such that higher scores indicate more of each construct. Scores of 4 or higher on the emotional exhaustion and depersonalization subscales indicate a greater burden of burnout symptoms.

We assessed the work engagement of survey respondents using the Utrecht Work Engagement Scale, which consists of 17 items across three dimensions (dimension one, vitality; dimension two, dedication; and dimension three, focus), with higher scores (greater than or equal to 4) indicating higher levels of work engagement [13].

Participants

The participants were recruited using convenience sampling among trauma clinicians at the QEII in Halifax, Nova Scotia. The survey was administered to all Trauma Team Leaders (n=19, including seven who are also Trauma Consult Physicians) and Trauma Consult Nurses (n=5) at a level 1 trauma center. Therefore, the maximum possible number of participants was 24, and the maximum possible number of responses across all three points was 72. The trauma center and TCS employ a mixed-model care approach, in which both the Trauma Team Leader and the Trauma Consult Physician may be surgeons or non-surgeons.

Statistical analysis

Patient demographics and characteristics were reported with descriptive statistics. To maintain privacy, cell sizes less than five are reported as "n<5," with adjacent cells reported as "n>5." Generalized estimating equations (GEE) were fitted to determine the effect of implementing the TCS on burnout symptoms. Generalized estimating equations (GEE) are a statistical method for analyzing correlated data, such as repeated measurements on the same subjects across measurement occasions. Generalized estimating equations account for this correlation, providing more accurate estimates of relationships between variables. We used generalized estimating equations to compare mean scores on each burnout scale.

A sensitivity analysis was conducted because only 10 participants responded to the survey at two or more time points (<5 responded at all three time points), yielding both paired and unpaired data. All survey responses were included in the analysis, as excluding participants with missing data may introduce potential selection bias. The sensitivity analysis consisted of a GEE model with independence correction and two linear models: a pooled and a mixed-effects model.

## Results

There were 16 participants out of a possible 24 (66.7% participation rate). Of the participants, 62.5% (10/16) responded at multiple time points (<5 responded at all three time points), yielding a cumulative response rate of 40.3% (29/72) across all three time points. At each time point, the response rates were as follows: 54.2% (13/24) at zero months, 33.3% (8/24) at six months, and 33.3% (8/24) at 12 months. Table 1 shows that the majority of responses across all three time points came from Trauma Team Leaders/Trauma Consult Physicians (n=13), followed by Trauma Team Leaders (n=9) and Trauma Consult Nurses (n=7). This is equivalent to a cumulative response rate of 38.6% (22/57) among physicians and 46.6% (7/15) among nurses. Nearly all were full-time employees (96.6%), and most had 6-10 years of experience providing trauma care (58.6%). The majority of the participants at zero months reported that COVID impacted their work at the initiation of the Trauma Consult Service (TCS) (64.6%), but this decreased to 13.3% at 12 months. Table 1 illustrates the self-reported impacts of work. Few participants reported using mental health resources to improve their well-being (3.4%).

**Table 1:** Participant characteristics at zero, six, and 12 months post-implementation of the Trauma Consult Service.

Characteristics		Overall (n=29)	0 months (n=13)	6 months (n=8)	12 months (n=8)
Age group in years, n (%)	31-40 years	14 (48.3)	6 (46.1)	n<5	n<5
	41-50 years	8 (27.6)	n<5	n<5	n<5
	>50 years	7 (24.1)	n<5	n<5	n<5
Sex, n (%)	Male	27 (93.1)	12 (92.3)	8 (100.0)	7 (87.5)
	Female	n<5	n<5	0 (0)	n<5
Position, n (%)	Trauma Team Leader/Trauma Consult Physician	13 (44.8)	5 (38.4)	n<5	n<5
	Trauma Team Leader	9 (31.0)	n<5	n<5	n<5
	Trauma Consult Nurse	7 (24.1)	n<5	n<5	n<5
Work status, n (%)	Full-time	28 (96.6)	13 (0.0)	7 (87.5)	8 (100.0)
	Part-time	n<5	0 (0)	n<5	0 (0)
Years providing trauma care, n (%)	<1-5 years	6 (20.7)	n<5	n<5	n<5
	6-10 years	17 (58.6)	7 (53.8)	n>5	n<5
	>10 years	6 (20.7)	n<5	n<5	n<5
Percent of work time involving trauma care, n (%)	0%-20%	13 (44.8)	6 (46.2)	n<5	n<5
	21%-40%	6 (20.7)	n<5	n<5	n<5
	41%-60%	6 (20.7)	n<5	n<5	n<5
	61%-80%	2 (6.9)	0 (0)	n<5	n<5
	81%-100%	2 (6.9)	0 (0)	n<5	n<5
Have children living at home, n (%)		18 (62.1)	7 (53.8)	6 (75.0)	5 (62.5)
Had COVID in the past year, n (%)		15 (51.7)	10 (76.9)	n<5	n<5
Missed work due to COVID, n (%)		17 (58.6)	11 (84.6)	n<5	n<5
Approach to work was dramatically impacted by COVID, n (%)		13 (44.8)	8 (61.5)	n<5	n<5
Total COVID impact, n (%)		45 (51.7)	29 (64.4)	10 (22.2)	6 (13.3)
Recipient of physical abuse, n (%)		8 (27.6)	5 (38.5)	n<5	n<5
Recipient of bullying, n (%)		10 (34.5)	5 (38.5)	n<5	n<5
Recipient of verbal abuse, n (%)		20 (69.0)	10 (76.9)	5 (62.5)	5 (62.5)
Recipient of a lack of care, n (%)		13 (44.8)	7 (53.8)	n<5	n<5
Recipient of a lack of respect, n (%)		21 (72.4)	9 (69.2)	6 (75.0)	6 (75.0)
Recipient of a lack of trust, n (%)		15 (51.7)	5 (38.5)	5 (62.5)	5 (62.5)
Total negative experiences reported, n (%)		79 (45.4)	36 (45.6)	24 (30.4)	19 (24.1)
Used mental health resources to improve well-being, n (%)		n<5	n<5	0 (0)	0 (0)

The GEE models for the Copenhagen Burnout Inventory, Maslach Burnout Inventory, and Utrecht Work Engagement Scales are summarized in Table 2. The sensitivity analysis shows consistency when the data are modelled under different assumptions (see Appendices). Trauma clinicians reported a decrease in burnout levels and an increase in engagement levels at the one-year follow-up. Generalized estimating equations demonstrate that overall, there was a downward trend in burnout symptoms and an increase in work engagement.

**Table 2:** Effect of the Trauma Consult Service on staff burnout levels at one-year follow-up. Data are presented at each time point as mean±standard deviation. Copenhagen Burnout Inventory (range: zero to five; scores greater than or equal to 3 indicate a high degree of burnout); Maslach Burnout Inventory (range: zero to six; higher scores indicate higher burnout levels); Utrecht Work Engagement Scale (range: zero to six; higher scores predict greater engagement and reduced risk of burnout). Cronbach's alpha is a measure of internal consistency (values of ≥0.7 indicate acceptable internal consistency). OR, odds ratio; CI, confidence interval

Assessment tool		0 months (n=13)	6 months (n=8)	12 months (n=8)	OR	95% CI	P value	Cronbach's alpha
Copenhagen Burnout Inventory	Personal burnout	3.1±0.9	2.7±0.8	2.2±0.6	0.93	0.89-0.97	<0.001	0.91
	Work-related burnout	3.4±1.0	2.9±0.6	2.5±1.1	0.93	0.87-0.99	0.016	0.94
	Patient-related burnout	2.6±1.1	2.4±0.8	1.8±0.7	0.94	0.88-1.00	0.07	0.927
	Overall score	3.3±0.9	2.7±0.7	2.2±0.6	0.93	0.88-0.98	0.012	0.968
Maslach Burnout Inventory single-item measures	Emotional exhaustion	3.8±1.6	2.6±2.1	2.5±1.7	0.88	0.79-0.98	0.023	-
	Depersonalization	4.2±1.7	3.0±1.5	2.4±1.8	0.86	0.76-0.98	0.023	-
Utrecht Work Engagement Scale	Vitality/vigor	3.6±1.2	4.3±0.5	5.0±0.4	1.12	1.06-1.19	<0.001	0.9
	Dedication	4.2±1.0	4.5±0.7	5.2±0.6	1.08	1.02-1.14	0.005	0.866
	Focus/absorption	4.1±1.0	4.1±0.6	4.8±0.5	1.06	1.01-1.11	0.019	0.721
	Overall score	3.9±1.0	4.3±0.6	5.0±0.4	1.09	1.03-1.14	0.001	0.937

**Figure 1:**
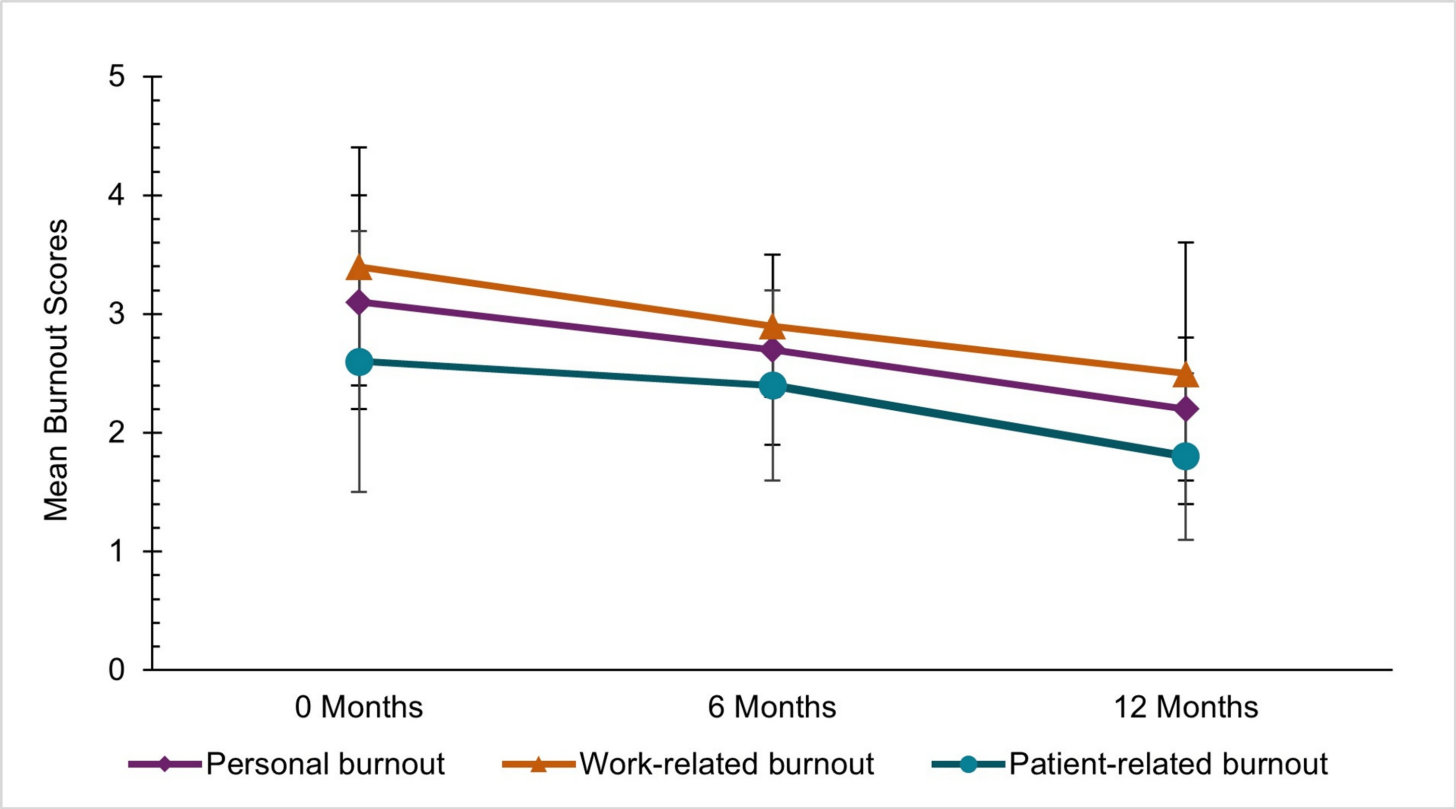
Burnout symptom scores measured by the Copenhagen Burnout Inventory at zero, six, and 12 months. The Copenhagen Burnout Inventory includes three subscales: personal burnout, work-related burnout, and patient-related burnout. Scores less than 2 indicate low levels of burnout, and scores greater than or equal to 3 indicate a high degree of burnout.

There were reductions in burnout demonstrated in the Copenhagen Burnout Inventory scores between zero and 12 months (odds ratio {OR}, 0.93; 95% confidence interval {CI}, 0.88-0.99) (Figure 1). Both personal (3.1±0.9) and work-related (3.4±1.0) burnout at zero months are both scores over 3, indicating high burnout. All Copenhagen Burnout Inventory measures at 12 months decreased to less than three, indicating low to intermediate burnout (overall=2.2±0.6). Notably, the decrease in personal burnout (OR, 0.93; 95% CI, 0.89-0.97) was significant (p-value<0.001). There were also significant decreases in work-related (OR, 0.93; 95% CI, 0.87-0.99) and patient-related (OR, 0.94; 95% CI, 0.87-1.99) burnout. The internal consistency of the Copenhagen Burnout Inventory measures was high (overall Cronbach's α=0.968).

**Figure 2:**
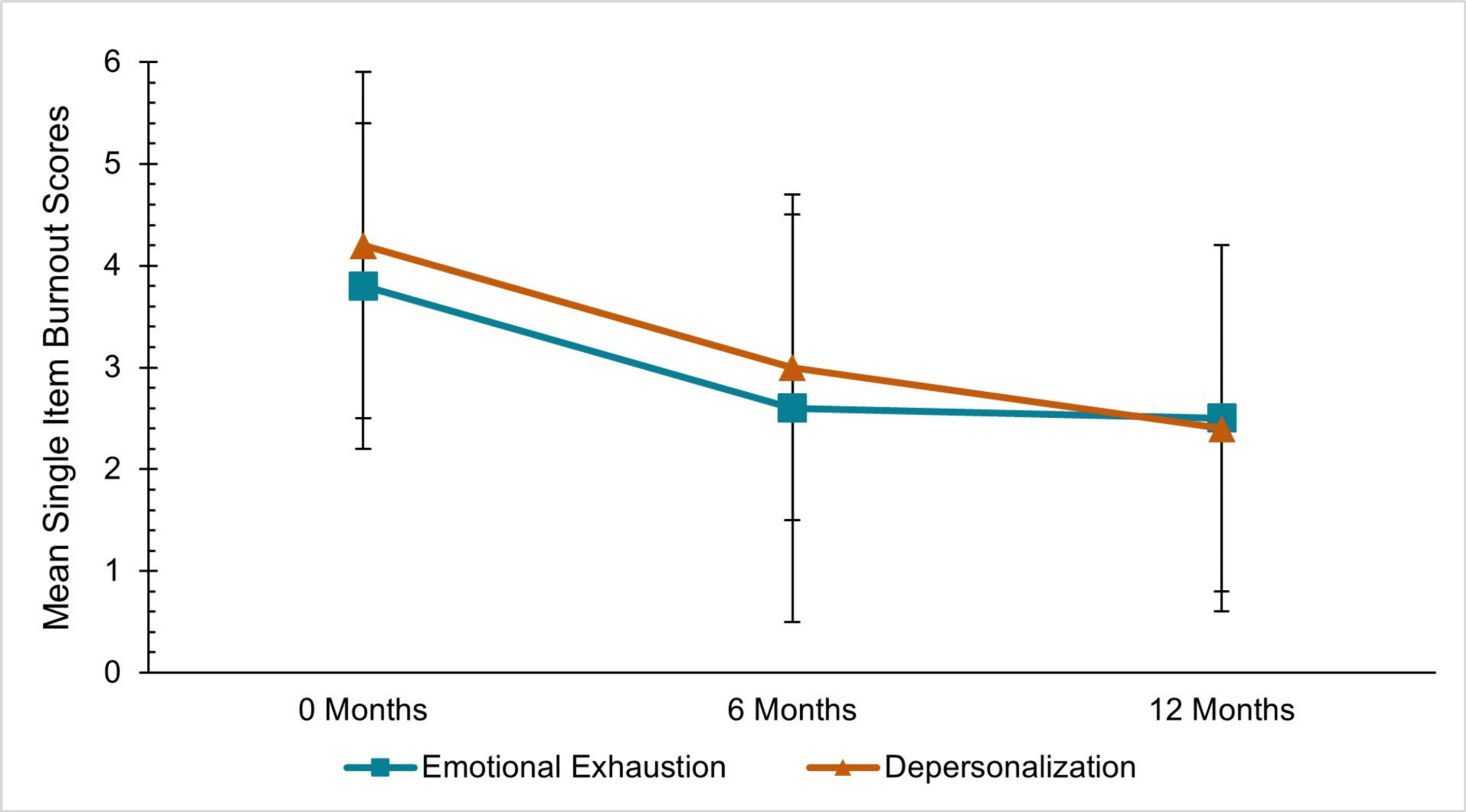
Maslach Burnout Inventory single-item scale scores for emotional exhaustion and depersonalization at zero, six and 12 months. Scores greater than or equal to 4 indicate a greater burden of burnout symptoms.

For the Maslach Burnout Inventory, each of the single-item measures showed decreases in emotional exhaustion and depersonalization symptoms (Figure 2). At zero months, emotional exhaustion scores (3.8±1.6) decreased at 12 months (2.5±1.7). Similarly, for depersonalization, the score at zero months (4.2±1.7) decreased to 12 months (2.4±1.8). For emotional exhaustion (OR, 0.88; 95% CI, 0.79-0.98) and depersonalization (OR, 0.86; 95% CI, 0.76-0.98), the generalized estimating equations both showed a significant decrease from zero to 12 months, with p-values of 0.023.

**Figure 3:**
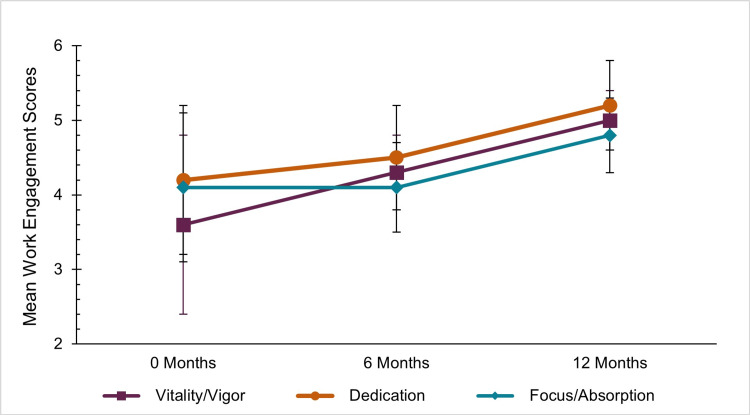
Utrecht Work Engagement Scale scores at zero, six, and 12 months. The Utrecht Work Engagement Scale includes three subscales: vigor, dedication, and focus/absorption. Scores greater than or equal to 4 indicate higher levels of work engagement.

There was also increased staff engagement across all three Utrecht Work Engagement Scales (overall score: OR, 1.09; 95% CI, 1.03-1.14) (Figure 3). Specifically, the vigor scores increased from zero months (3.6±1.2) to 12 months (5.0±0.4), which showed a significant effect with a p-value of <0.001 (OR, 1.12; 95% CI, 1.06-1.19). Additionally, we noted substantial increases in dedication (OR, 1.08; 95% CI, 1.02-1.14) and focus/absorption (OR, 1.06, 95%CI 1.01-1.11). The Utrecht Work Engagement Scales measured high internal consistency (overall Cronbach's α: 0.937).

## Discussion

Statement of principal findings

Our study found an association of reduced burnout symptoms (as measured by the Copenhagen Burnout Inventory, the Maslach Burnout Inventory, and the Utrecht Work Engagement Scale) among clinicians who worked for the novel Trauma Consult Service (TCS) over 12 months. The Copenhagen Burnout Inventory indicated reductions in both personal and work-related burnout. At the same time, the Maslach Burnout Inventory demonstrated reductions in emotional exhaustion and depersonalization, and the Utrecht Work Engagement Scale revealed increased work engagement. Clinicians (physicians and nurses) who initiated a change in their clinical focus (in part or in total) to include the resuscitation and longitudinal care of major trauma patients all reported significant improvement in all burnout measures, consistent with improved clinician well-being. Our results suggest that systemic changes, such as the introduction of a novel TCS, can support career redefinition and are associated with an improvement in population-level clinician resilience and job satisfaction. Features of workplace happiness and engagement include clinicians who focus on novel, challenging, and personally meaningful work [14].

This study also found reductions in the impact COVID had on staff over the 12-month period. Therefore, the TCS shows promise in reducing burnout. However, the decrease in burnout systems could be attributed to other factors, such as the decreasing COVID burden. The evaluation of system performance demonstrated that the TCS improved patient, provider, and family satisfaction, which may be important variables contributing to the improvement in our physicians' and nurses' burnout symptoms [15]. The TCS has been recognized locally and nationally for its excellence in quality of care and patient experience, which underscores the importance of the TCS to trauma care in Nova Scotia and the Maritime region [16].

Interpretation within the context of the wider literature

Similar studies conducted internationally and in Canada have not found a link between burnout and the establishment of an inpatient TCS [6,8]. A Canadian survey of emergency medicine burnout during the early COVID pandemic found that team support and access to resources were key in reducing burnout [7]. This phenomenon has also been observed in the United States and Europe, both before and during the COVID pandemic [17,18]. Organizational changes that reduce clinical burden while maximizing efficiency are viable options for reducing burnout [19,20]. Investigators recommend engaging providers before implementing organizational changes to allow them to bring forward suggestions that apply to the local setting [21]. In these cases, changes introduced have limited the unpredictability of clinical workload and patient acuity, ultimately reducing burnout [22]. Alternatively, moving from 24-hour call to 12-hour blocks improved burnout among staff without compromising clinical productivity [23].

Our study focused on a partial career shift as the intervention itself. In contrast, other studies have explored wellness interventions, such as mindfulness-based programs and those improving communication and physician well-being [24]. Wellness leadership interventions have been shown to decrease burnout symptoms, as demonstrated in a six-week program in the provincial health system in Nova Scotia [2]. Other wellness interventions that may be effective in decreasing burnout symptoms include regular physical activity, healthy dietary habits, social engagement, mentorship programs, and regular health checkups [25].

Interventions to reduce burnout symptoms have demonstrated some effectiveness, including adjustments to job demands, changes in work structure, and physical activity initiatives [23,26]. Our investigation suggests that organizational changes may impact burnout symptoms, especially when clinicians find renewed purpose and satisfaction in their work. In this study, a system-level change was introduced (i.e., the new TCS), which enabled trauma clinicians to refocus their clinical work, transitioning in part from their base specialty to trauma resuscitation combined with inpatient consulting. This change resulted in a positive impact on their burnout levels within 12 months. As healthcare systems evolve to support optimal patient care, prioritizing clinician well-being through such role shifts when viable is essential to optimize both patient care and career satisfaction. However, the specific reasons for this reduction remain unclear. Improvement in burnout may have been due to a combination of role changes, team dynamics, or other factors. Clinicians in this study experienced new roles and/or career change, and it should be explored whether these changes are transferable to other trauma centers and clinicians.

Strengths and limitations

This study has several strengths. First, it offers a longitudinal analysis of burnout symptoms over a 12-month period, which allows insight into the impact of the TCS. The use of multiple validated burnout and engagement measures enhances the robustness of our findings, which can be compared to a wide range of studies that used the same measures [10,11,13]. Additionally, the diverse sample of Trauma Team Leaders and Nurses from different disciplines added to the generalizability of the results within similar high-stress healthcare settings.

Limitations to consider include a limited sample size due to the select group of clinicians involved with the TCS. While the cumulative response rate for all possible respondents across all time points was 40%, only eight respondents were included in six months and eight in 12 months. Only 10 participants responded to the survey at two or more time points, with <5 responding at all three time points. While only 24.1% (7/29) of responses were from nurses, 20% of the trauma staff are nurses, making the response rate proportional. We also relied on self-reported measures, which may be susceptible to response and recall biases. As the number of responses decreased for the subsequent time points (six and 12 months), there could also be bias, as clinicians with less burnout were more likely to respond to the survey. We reported burnout levels at all time points, and the sensitivity analysis attests to the downward trend in burnout symptoms. However, the low response rate and incomplete follow-up at six and 12 months mean that the results reflect a population-level trend rather than a within-subject change. Further investigations are needed to affirm that the TCS did reduce burnout symptoms.

We did not explicitly ask our clinicians to explain why they believed they had burnout symptoms, which would be of interest in a future investigation. Multiple factors may have contributed to the clinician's burnout, including the fact that the TCS was initiated during the COVID pandemic, which likely had a significant impact. This makes COVID likely a confounding variable in the study. The study asked about COVID, including whether it had affected their work, and found a decrease in the percentage of respondents who answered yes compared to 12 months earlier. Because there was no control group, a comparison of burnout symptoms among different clinician groups was not evaluated. This addition in future studies could help determine if reductions were attributable to the TSC or other confounding factors, such as COVID. We also received the highest number of responses from Trauma Team Leaders/Trauma Consult Physicians, who would have been the ones to choose to change their practice to include inpatient care. This may have increased the chances of the burnout symptoms decreasing, because they actively decided to change their practice. Although we believe that the TCS contributed to the reported improvement in burnout scores, it is possible that the lessening impact of COVID or other factors could be cofounding variables.

Implications for policy, practice, and research

The reduction in burnout symptoms among TCS clinicians is promising, but long-term sustainability remains uncertain. Investigating burnout symptoms at the initiation of the TCS was essential for understanding its impact. Other studies found that burnout levels increased post-pandemic, therefore requiring ongoing assessments to determine whether our TCS can sustain its positive impact on burnout symptoms. Follow-up studies that address the limitations of a low response rate, COVID's impact, and the lack of a control group would be beneficial for continued evaluations. Continued evaluation is crucial to ensure that clinicians receive adequate support and to explore how systemic changes can have a lasting time-related association with clinician well-being. Additionally, investigating barriers to mental health resource utilization and implementing targeted interventions may lead to improved long-term outcomes. For example, mindfulness-based interventions have shown modest but sustained reductions in burnout [27]. In contrast, other researchers have focused on the qualitative features of addressing burnout, such as fostering healthy team environments, addressing toxic collegial behaviors, and helping staff process the emotions associated with being exposed to intense situations [14]. Further studies should explore the impact of the TCS on patient outcomes, as organizational strategies, such as those summarized by Zhang et al. (2022), suggest that bundled interventions may be most effective [28].

## Conclusions

Over 12 months, the implementation of a dedicated inpatient TCS was associated with a reduction in burnout symptoms and increased engagement among trauma clinicians. Further investigation is warranted to confirm whether these findings are attributable to a change in practice rather than to other factors, such as the COVID pandemic. The critical need to address burnout among emergency medicine staff globally cannot be overstated, as improving clinician well-being ultimately improves care and the functioning of healthcare systems as a whole.

## Appendices

Table 3 shows the burnout in the trauma survey.

**Table 3:** Burnout in the trauma survey. *Items are protected by copyright.

Part A: demographics		
Questions		Response options
Q1. What is your age?		20-30 years
		31-40 years
		41-50 years
		51-60 years
		>60 years
Q2. What is your sex?		Male
		Female
		Prefer not to say
		Others, please specify: _______________
Q3. What is your position?		Physician
		Nurse
		Others, please specify: _______________
Q4. Do you have any children living at home?		Yes
		No
		Prefer not to say
Q5. What is your work status?		Full-time
		Part-time
		Others, please specify: _______________
Q6. How long have you worked in trauma care?		<1 year
		1-2 years
		3-5 years
		6-10 years
		11-15 years
		16-20 years
		>20 years
Q7. Have you had COVID in the past year?		Yes
		No
		Prefer not to say
Q8. Did you miss work from COVID?		Yes
		No
		Prefer not to say
Q9. Has COVID had a dramatic effect on how you approach your work?		Yes
		No
		Prefer not to say
Q10. What proportion of your time at work do you estimate involves caring for trauma patients?		0%-20%
		21%-40%
		41%-60%
		61%-80%
		81%-100%
Q11. In the past six months, have you personally been the recipient of any of the following while you were providing trauma care? (Yes/no/prefer not to say)	Physical violence	Yes
		No
		Prefer not to say
	Bullying	Yes
		No
		Prefer not to say
	Verbal abuse	Yes
		No
		Prefer not to say
	Lack of care	Yes
		No
		Prefer not to say
	Lack of respect	Yes
		No
		Prefer not to say
	Lack of trust	Yes
		No
		Prefer not to say
Q12. In the past six months, have you used any mental health resources to improve your well-being?		Yes (if "yes," the participants were asked to specify which resource(s) they used)
		No
Part B: Utrecht Work Engagement Scale (UWES)		
Q13. The following 17 statements are about how you feel at work. Please read each statement carefully and decide if you ever feel this way about your job. If you have had this feeling, indicate how often you feel it by selecting the number from 1 (almost never) to 6 (always) that best describes how frequently you feel that way. If you have never had this feeling, select "0"		
Statements		Response options
1. At my work, I feel bursting with energy		0. Never
		1. Almost never (a few times a year or less)
		2. Rarely (once a month or less)
		3. Sometimes (a few times a month)
		4. Often (once a week)
		5. Very often (a few times a week)
		6. Always (every day)
2. I find the work that I do full of meaning and purpose		0. Never
		1. Almost never (a few times a year or less)
		2. Rarely (once a month or less)
		3. Sometimes (a few times a month)
		4. Often (once a week)
		5. Very often (a few times a week)
		6. Always (every day)
3. Time flies when I am working		0. Never
		1. Almost never (a few times a year or less)
		2. Rarely (once a month or less)
		3. Sometimes (a few times a month)
		4. Often (once a week)
		5. Very often (a few times a week)
		6. Always (every day)
4. At my job, I feel strong and vigorous		0. Never
		1. Almost never (a few times a year or less)
		2. Rarely (once a month or less)
		3. Sometimes (a few times a month)
		4. Often (once a week)
		5. Very often (a few times a week)
		6. Always (every day)
5. I am enthusiastic about my job		0. Never
		1. Almost never (a few times a year or less)
		2. Rarely (once a month or less)
		3. Sometimes (a few times a month)
		4. Often (once a week)
		5. Very often (a few times a week)
		6. Always (every day)
6. When I am working, I forget everything else around me		0. Never
		1. Almost never (a few times a year or less)
		2. Rarely (once a month or less)
		3. Sometimes (a few times a month)
		4. Often (once a week)
		5. Very often (a few times a week)
		6. Always (every day)
7. My job inspires me		0. Never
		1. Almost never (a few times a year or less)
		2. Rarely (once a month or less)
		3. Sometimes (a few times a month)
		4. Often (once a week)
		5. Very often (a few times a week)
		6. Always (every day)
8. When I get up in the morning, I feel like going to work		0. Never
		1. Almost never (a few times a year or less)
		2. Rarely (once a month or less)
		3. Sometimes (a few times a month)
		4. Often (once a week)
		5. Very often (a few times a week)
		6. Always (every day)
9. I feel happy when I am working intensely		0. Never
		1. Almost never (a few times a year or less)
		2. Rarely (once a month or less)
		3. Sometimes (a few times a month)
		4. Often (once a week)
		5. Very often (a few times a week)
		6. Always (every day)
10. I am proud of the work that I do		0. Never
		1. Almost never (a few times a year or less)
		2. Rarely (once a month or less)
		3. Sometimes (a few times a month)
		4. Often (once a week)
		5. Very often (a few times a week)
		6. Always (every day)
11. I am immersed in my work		0. Never
		1. Almost never (a few times a year or less)
		2. Rarely (once a month or less)
		3. Sometimes (a few times a month)
		4. Often (once a week)
		5. Very often (a few times a week)
		6. Always (every day)
12. I can continue working for very long periods at a time		0. Never
		1. Almost never (a few times a year or less)
		2. Rarely (once a month or less)
		3. Sometimes (a few times a month)
		4. Often (once a week)
		5. Very often (a few times a week)
		6. Always (every day)
13. To me, my job is challenging		0. Never
		1. Almost never (a few times a year or less)
		2. Rarely (once a month or less)
		3. Sometimes (a few times a month)
		4. Often (once a week)
		5. Very often (a few times a week)
		6. Always (every day)
14. I get carried away when I am working		0. Never
		1. Almost never (a few times a year or less)
		2. Rarely (once a month or less)
		3. Sometimes (a few times a month)
		4. Often (once a week)
		5. Very often (a few times a week)
		6. Always (every day)
15. At my job, I am very resilient, mentally		0. Never
		1. Almost never (a few times a year or less)
		2. Rarely (once a month or less)
		3. Sometimes (a few times a month)
		4. Often (once a week)
		5. Very often (a few times a week)
		6. Always (every day)
16. It is difficult to detach myself from my job		0. Never
		1. Almost never (a few times a year or less)
		2. Rarely (once a month or less)
		3. Sometimes (a few times a month)
		4. Often (once a week)
		5. Very often (a few times a week)
		6. Always (every day)
17. At my work, I always persevere, even when things do not go well		0. Never
		1. Almost never (a few times a year or less)
		2. Rarely (once a month or less)
		3. Sometimes (a few times a month)
		4. Often (once a week)
		5. Very often (a few times a week)
		6. Always (every day)
Part C: single-item measures of emotional exhaustion and depersonalization		
Q14. Please read the following two statements carefully and decide how often you feel this way about your job		
Statements		Response options
1. Emotional exhaustion statement*		1. Never
		2. A few times a year
		3. A few times a month
		4. A few times a week
		5. Once a week
		6. A few times a week
		7. Every day
2. Depersonalization statement*		1. Never
		2. A few times a year
		3. A few times a month
		4. A few times a week
		5. Once a week
		6. A few times a week
		7. Every day
Part D: Copenhagen Burnout Inventory (CBI)		
Q15. Personal burnout is a state of prolonged physical and psychological exhaustion. Please answer the following questions regarding personal burnout		
Questions		Response options
1. How often do you feel tired?		1. Never/almost never
		2. Seldom
		3. Sometimes
		4. Often
		5. Always
2. How often are you physically exhausted?		1. Never/almost never
		2. Seldom
		3. Sometimes
		4. Often
		5. Always
3. How often are you emotionally exhausted?		1. Never/almost never
		2. Seldom
		3. Sometimes
		4. Often
		5. Always
4. How often do you think, "I can't take it anymore"?		1. Never/almost never
		2. Seldom
		3. Sometimes
		4. Often
		5. Always
5. How often do you feel worn out?		1. Never/almost never
		2. Seldom
		3. Sometimes
		4. Often
		5. Always
6. How often do you feel weak and susceptible to illness?		1. Never/almost never
		2. Seldom
		3. Sometimes
		4. Often
		5. Always
Q16. Work-related burnout is a state of prolonged physical and psychological exhaustion, which is perceived as related to the person's work. Please answer the following questions regarding work-related burnout		
Questions		Response options
1. Is your work emotionally exhausting?		1. To a very low degree
		2. To a low degree
		3. Somewhat
		4. To a high degree
		5. To a very high degree
2. Do you feel burnt out because of your work?		1. To a very low degree
		2. To a low degree
		3. Somewhat
		4. To a high degree
		5. To a very high degree
3. Does your work frustrate you?		1. To a very low degree
		2. To a low degree
		3. Somewhat
		4. To a high degree
		5. To a very high degree
4. Do you feel worn out at the end of the working day?		1. Never/almost never
		2. Seldom
		3. Sometimes
		4. Often
		5. Always
5. Are you exhausted in the morning at the thought of another day at work?		1. Never/almost never
		2. Seldom
		3. Sometimes
		4. Often
		5. Always
6. Do you feel that every working hour is tiring for you?		1. Never/almost never
		2. Seldom
		3. Sometimes
		4. Often
		5. Always
7. Do you have enough energy for family and friends during leisure time?		1. Never/almost never
		2. Seldom
		3. Sometimes
		4. Often
		5. Always
Q17. Patient-related burnout is a state of prolonged physical and psychological exhaustion, which is perceived as related to the person's work with patients. Please answer the following questions regarding patient-related burnout		
Questions		Response options
1. Do you find it hard to work with patients?		1. To a very low degree
		2. To a low degree
		3. Somewhat
		4. To a high degree
		5. To a very high degree
2. Do you find it frustrating to work with patients?		1. To a very low degree
		2. To a low degree
		3. Somewhat
		4. To a high degree
		5. To a very high degree
3. Does it drain your energy to work with patients?		1. To a very low degree
		2. To a low degree
		3. Somewhat
		4. To a high degree
		5. To a very high degree
4. Do you feel that you give more than you get back when you work with patients?		2. To a low degree
		3. Somewhat
		4. To a high degree
		5. To a very high degree
5. Are you tired of working with patients?		1. Never/almost never
		2. Seldom
		3. Sometimes
		4. Often
		5. Always
6. Do you sometimes wonder how long you will be able to continue working with patients?		1. Never/almost never
		2. Seldom
		3. Sometimes
		4. Often
		5. Always
Q18. If you have additional comments about burnout, please enter them here		(Free text)

Table 4 shows the sensitivity analysis for the effects of the Trauma Consult Service on burnout levels.

**Table 4:** Sensitivity analysis for the effects of the Trauma Consult Service on burnout levels.

Assessment tool			OR	95% CI	P-value
Copenhagen Burnout Inventory	Personal burnout	Generalized estimating equation	0.93	0.89-0.97	<0.001
		Generalized estimating equation with independence	0.93	0.89-0.97	0.001
		Pooled linear model	0.93	0.88-0.99	0.020
		Mixed-effects model	0.93	0.89-0.97	<0.001
	Work-related burnout	Generalized estimating equation	0.93	0.87-0.99	0.016
		Generalized estimating equation with independence	0.93	0.87-0.99	0.017
		Pooled linear model	0.93	0.87-0.99	0.022
		Mixed-effects model	0.93	0.88-0.98	0.010
	Patient-related burnout	Generalized estimating equation	0.94	0.88-1.00	0.07
		Generalized estimating equation with independence	0.94	0.88-1.01	0.084
		Pooled linear model	0.94	0.88-1.01	0.113
		Mixed-effects model	0.95	0.89-1.00	0.068
	Overall score	Generalized estimating equation	0.93	0.88-0.98	0.012
		Generalized estimating equation with independence	0.93	0.88-0.99	0.013
		Pooled linear model	0.93	0.88-0.99	0.028
		Mixed-effects model	0.94	0.89-0.98	0.009
Maslach Burnout Inventory single-item measures	Emotional exhaustion	Generalized estimating equation	0.88	0.79-0.98	0.023
		Generalized estimating equation with independence	0.89	0.79-1.00	0.048
		Pooled linear model	0.89	0.78-1.01	0.084
		Mixed-effects model	0.89	0.79-1.00	0.048
	Depersonalization	Generalized estimating equation	0.86	0.76-0.98	0.023
		Generalized estimating equation with independence	0.86	0.75-0.98	0.026
		Pooled linear model	0.86	0.76-0.97	0.021
		Mixed-effects model	0.86	0.76-0.97	0.014
Utrecht Work Engagement Scale	Vitality/vigor	Generalized estimating equation	1.12	1.06-1.19	<0.001
		Generalized estimating equation with independence	1.12	1.06-1.19	<0.001
		Pooled linear model	1.12	1.05-1.20	0.002
		Mixed-effects model	1.12	1.05-1.19	<0.001
	Dedication	Generalized estimating equation	1.08	1.02-1.14	0.005
		Generalized estimating equation with independence	1.08	1.02-1.14	0.005
		Pooled linear model	1.08	1.02-1.15	0.014
		Mixed-effects model	1.08	1.02-1.14	0.005
	Focus/absorption	Generalized estimating equation	1.06	1.01-1.11	0.019
		Generalized estimating Equation with independence	1.06	1.01-1.11	0.017
		Pooled linear model	1.06	1.00-1.13	0.061
		Mixed-effects model	1.06	1.01-1.11	0.021
	Overall score	Generalized estimating equation	1.09	1.03-1.14	0.001
		Generalized estimating equation with independence	1.09	1.03-1.14	0.001
		Pooled linear model	1.09	1.03-1.15	0.007
		Mixed-effects model	1.09	1.03-1.14	0.002
